# Biomarker Profiling of Cardiac Causes for Chest Pain in Non-acute Coronary Syndrome Patients in West Virginia

**DOI:** 10.7759/cureus.104002

**Published:** 2026-02-20

**Authors:** Sneha S Pillai, Muhammad A Chaudhry, Bruno De Souza Goncalves, Izza Saeed, Hibba Chaudhry, Mahir Irtiza, Charles J Williams, Ji Bihl, Alip Borthakur, Ellen Thompson, Komal Sodhi

**Affiliations:** 1 Surgery and Biomedical Sciences, Marshall University Joan C. Edwards School of Medicine, Huntington, USA; 2 Cardiology, Marshall University Joan C. Edwards School of Medicine, Huntington, USA; 3 Medical School, West Virginia University School of Medicine, Morgantown, USA; 4 Biological Sciences, West Virginia University, Morgantown, USA; 5 Dornsife Department of Letters and Sciences, University of Southern California, Los Angeles, USA; 6 Biomedical Sciences, Marshall University Joan C. Edwards School of Medicine, Huntington, USA

**Keywords:** acute coronary syndrome, biomarkers, cardiac dysfunction, prognosis, risk stratification

## Abstract

Introduction

The lifetime risk of developing acute coronary syndrome (ACS) is heavily influenced by modifiable factors like smoking, hypertension, diabetes, and lifestyle, as well as non-modifiable factors such as age, sex, and genetic predisposition. As the primary symptom of ACS, chest pain accounts for the most common reasons for emergency department visits and outpatient cardiac evaluations in the United States, posing a significant diagnostic challenge to clinicians. ACS carries a massive economic burden, with high direct costs from hospitalizations and high indirect costs like lost productivity, impacting healthcare systems, especially in rural areas like West Virginia, with a high prevalence of risk factors associated with cardiovascular disease progression. Circulating biomarkers present a promising approach in the research and clinical practice of various diseases as they are minimally invasive, highly cost-effective, and provide high specificity. So, the objective of the present study was to evaluate the potential of a biomarker panel in differentiating cardiac causes of chest pain in non-ACS patients in West Virginia.

Methods

The exploratory cross-sectional study was conducted in patients from a hospital in West Virginia, United States. Patients who were referred to the Rapid Access Chest Pain Clinic were enrolled. Patients were selected based on the established inclusion and exclusion criteria. The plasma samples from chest pain patients, in comparison with age-matched controls, were used for the assessment of circulating biomarkers of cardiac dysfunction such as N-terminal pro-B-type natriuretic peptide (NT-proBNP), Myosin-binding protein C (MyBP-C), creatine kinase-myocardial Band (CK-MB), and neopterin.

Results

Our results showed that there was a significant elevation in these biomarkers in patients with chest pain in comparison with healthy controls. The correlation analysis revealed a significant link between these parameters and NT-proBNP, the well-established and highly specific predictive markers of cardiac injury and cardiac function decline. This multimarker approach that assessed the additive value of the proposed biomarker analysis may provide an earlier assessment of overall patient risk and may aid in identifying patients with a higher risk of an adverse event.

Conclusion

The findings in the present study demonstrate the potential of the four-biomarker panel in identifying the cardiac causes of chest pain and the future translational applicability of the proposed biomarker panel to determine and compare the prognostic abilities for early ACS detection through detailed longitudinal studies. This may also help in diagnosis, risk stratification, and guidance of treatment, further allowing management of cardiac function decline and improved health outcomes in the high-risk population of West Virginia.

## Introduction

Acute coronary syndrome (ACS) is a leading cause of global morbidity and mortality that represents a life-threatening spectrum of diseases caused by the compromised blood flow to the coronary vasculature, resulting in myocardial ischemia and infarction [1]. Both genetic and lifestyle factors contribute to this disease pathology, in which smoking, hypertension, diabetes, hyperlipidemia, sedentary lifestyle, poor food habits, and family history act as common risk factors [2]. ACS is the manifestation of atherosclerotic plaque that consists of various dynamic processes such as inflammatory progression, immunological reactions, oxidative stress, endothelial dysfunction, and dyslipidemia [3]. The progression of atherosclerotic plaque involves the gradual accumulation of lipids, inflammatory mediators, and debris in artery walls, transforming into vulnerable lesions [4]. The gradual progression of atherosclerotic plaque causes severe vascular narrowing and arterial blockage, followed by fibrous cap rupture and thrombus formation, which triggers ACS by blocking blood flow to the heart [5,6].

Chest pain is the most frequently reported symptom of ACS and one of the most common reasons for emergency department visits and outpatient cardiac evaluations in the United States (US), posing a significant diagnostic challenge to clinicians [7]. As any inaccurate risk stratification of chest pain can result in serious morbidity and mortality from ACS and other serious pathologies, the prompt and precise identification of cardiac causes of chest pain is critical for improved patient outcomes. Despite recent progress in the field of cardiovascular disease, the burden of disease, complementing ACS, remains extensive.

ACS poses a substantial economic burden to the US healthcare system with high direct costs from hospitalizations and other significant indirect costs [8,9]. The disease burden of ACS is pronounced in areas with high prevalence of risk factors associated with cardiovascular disease progression. The reports of the West Virginia Department of Health point out the highest rates of smoking, obesity, hypertension, diabetes, and physical inactivity in the population, all of which substantially increase lifetime cardiovascular risk [10]. With the increased prevalence of chronic metabolic diseases that significantly increase the risk of ACS-associated complications, the rural population of West Virginia faces a substantial economic burden, disproportionate health, and socioeconomic challenges. This emphasizes the need for region-specific research aimed at improving early detection and management strategies for systematic monitoring to minimize the risk of progression of cardiovascular complications in this population.

The metabolic and molecular anomalies in various disease pathologies can be reflected in circulation through specific biomarkers [11]. Circulating biomarkers offer a promising strategy in both research and clinical practice for numerous chronic diseases, as they are minimally invasive, cost-effective, and exhibit high specificity, providing significant advantages over traditional invasive procedures [12-15]. The remarkable correlation between biomarkers and disease progression provides opportunities for personalized medicine through risk assessment, disease screening, prevention, and treatment. Of note, risk assessment is crucial for providing immediate attention and guiding effective medical therapy to reduce the risk of chronic cardiovascular events [16].

Numerous biomarkers from diverse pathophysiological pathways have been identified in association with cardiovascular complications to provide valuable prognostic information, and incorporation of these circulating biomarkers into risk assessment may improve risk stratification [4,17-19]. Hence, it is integral to systematically evaluate the levels of these key biomarkers in the circulation of patients presenting with chest pain as indicators of myocardial injury and cardiac dysfunction, and it is critical to identify reliable biomarkers for early risk stratification of patients with chest pain.

Biomarkers such as N-terminal pro-B-type natriuretic peptide (NT-proBNP), myosin-binding protein C (MyBP-C), creatine kinase-myocardial band (CK-MB), and neopterin provide insight into key aspects of cardiac dysfunction, including myocardial wall stress, ventricular dysfunction, myocardial injury, immune activation, and myocardial necrosis [20-23]. A panel of biomarkers approach that combines established and emerging biomarkers may offer a more comprehensive understanding of cardiac pathology and aid in efficient early diagnosis to prevent future chronic cardiac diseases. Hence, the present pilot study aims to analyze a panel of reliable biomarkers that are related to cardiac dysfunction in patients with chest pain in the non-ACS population in West Virginia, with the goal of assessing their prognostic utility in the future through detailed longitudinal studies and improving outcomes in this high-risk population.

## Materials and methods

This study was conducted at the Cabell Huntington Hospital, Huntington, West Virginia, US. The study was approved by the Marshall University Institutional Review Board (reference number: RBNet ID# 1557770-16). The study was performed in accordance with the guidelines and regulations specified in the Declaration of Helsinki for the use and recruitment of human research subjects. Trained hospital personnel examined the medical records of patients with appropriate confidentiality measures in compliance with the Health Insurance Portability and Accountability Act (HIPAA), and ensured the suitable selection of patients eligible for the study. All patients who participated in the study were briefed about the use of blood samples for this research, and each patient voluntarily signed the informed consent form.

Study population

Patients who are referred to the Rapid Access Chest Pain Clinic at Cabell Huntington Hospital were enrolled in the study. Inclusion criteria were patients aged 18-70 years, presenting with chest pain, who had already had ACS excluded in the hospital. A standardized diagnostic protocol consistent with the guidelines of the American Heart Association (AHA) was used to exclude ACS in the chest pain population [24,25]. Briefly, all patients presenting with chest pain underwent comprehensive clinical assessments, including detailed history, cardiovascular risk evaluation, physical examination, high-sensitivity cardiac troponin (hs-cTn) measurement, and electrocardiograms (ECGs). ACS was excluded in the absence of any abnormal findings that mainly include persistent ischemic ECG changes and an hs-cTn value, which was confirmed by a board-certified cardiologist. The patients with age <18, whose risk of CAD is low, patients with age >70, whose risk of CAD is high, and those who were pregnant were excluded from the study.

Age-matched healthy subjects were enrolled as controls. A total of 40 subjects participated in the study, including 20 control subjects and 20 subjects with chest pain.

Blood collection and patient demographics

Blood was collected from all the patients recruited after receiving informed consent to participate in the study. The blood was collected in collection vials containing anticoagulant ethylenediaminetetraacetic acid (EDTA) and further processed for plasma as described previously [13-15,26]. Briefly, all blood samples were processed within 30 minutes of collection by centrifugation at 4,000 rpm for 10 minutes at 4°C. Following centrifugation, plasma was carefully separated and transferred into appropriately labeled tubes. Plasma samples were further divided to make aliquots of each sample to minimize repeated freeze-thaw cycles. All samples were stored at −80°C for analysis of biomarkers. Basic demographic characteristics, including age, gender, race/ethnicity, educational level, and socioeconomic status, were collected for all enrolled participants at the time of study enrollment.

Assessment of plasma biomarkers

Plasma samples stored at −80 °C were used for the assessment of biomarker levels by enzyme-linked immunosorbent assays (ELISA). The assays were performed in accordance with the manufacturers’ protocols using commercially available ELISA kits for each of the following biomarkers. Human NT-proBNP (Abcam Limited, Cambridge, United Kingdom), human myosin-binding protein C, cardiac-type (MyBioSource, Inc., San Diego, California, US), human CK-MB (Abcam Limited), and human neopterin (MyBioSource, Inc.). Briefly, assays were performed in 96-well plates according to the manufacturer's protocols. At the completion of each assay, absorbance was measured at 450 nm using a BioTek ELx800 microplate reader (Agilent Technologies, Inc., Santa Clara, California, US). A standard curve was plotted for each biomarker, and sample concentrations were calculated using the corresponding equations of the line of best fit. The detection limits, intra-assay coefficients of variation, and the specific ranges of the standard curves for each biomarker were based on the specific kit instructions.

Statistical analysis

The study was designed, conducted, analyzed, and interpreted in an unbiased manner to ensure reproducibility of the results. Statistical analyses were performed using GraphPad Prism version 10.0 (Dotmatics, Boston, Massachusetts, US). All data were tested for normality and then subjected to parametric analysis. The power analysis was performed to determine the adequate sample size for effective statistical comparison between groups. Briefly, a total sample size of 40 yielded approximately 80% power (Cohen’s d = 0.80) for a two-tailed t-test, an alpha level of 0.05, and equal allocation (1:1). An unpaired t-test with Welch's correction was performed. Statistical significance was defined as p < 0.05 or p < 0.01, corresponding to 95% or 99% confidence intervals, respectively. Data are presented as mean ± standard error of the mean (SEM). The correlation analysis was performed between NT-proBNP and other biomarkers analyzed, represented by plotting a scatter dot plot. The extent of correlation was determined by Pearson’s r coefficient using a 95% confidence interval and selected two-tailed P-value to determine significance (alpha=0.05) as described previously [13-15].

## Results

Baseline demographic and socioeconomic characteristics

Baseline demographic and socioeconomic characteristics were comparable between the control and chest pain populations. The comparable gender distribution between groups was evidenced by male patients representing 45% and 40% of participants in the control and chest pain groups, respectively, while female patients accounted for 55% and 60%, respectively. No significant changes were observed in the mean ages of both groups. The race and ethnicity of the study population were homogeneous, mainly consisting of non-Hispanic White patients. Even though it limits the applicability of the present findings in a more diverse population, it reflects the general demographic characteristics of the study recruitment area. Though educational levels were different between study populations, the socioeconomic status was comparable. Data for certain demographic characteristics were unavailable for some participants. The summary of the baseline demographic and socioeconomic characteristics of the study populations is presented in Table 1. 

**Table 1:** Baseline characteristics of study participants (N=40) ACS: acute coronary syndrome; SEM: standard error of the mean

Variable	Control Group (n=20)	Non-ACS Chest Pain Group (n=20)
Gender, n (%)		
Male	9 (45)	8 (40)
Female	11 (55)	12 (60)
Age (Years), mean ± SEM	57.30 ± 1.89	48.74 ± 4.26
	(Unpaired t test: ns, P=0.0783)	
Race/Ethnicity, n (%)		
Non-Hispanic White	15 (75)	19 (95)
Non-Hispanic Black	1 (5)	0
Data not Available	4 (20)	1 (5)
Educational Level (Year), n (%)		
≤9	2 (10)	0
10-12	4 (20)	1 (5 )
≥ 13	2 (10)	9 (45)
Data not Available	12 (60)	10 (50)
Social Economic Status, n (%)		
Low	9 (56.25)	10 (52.63)
Moderate/High	7 (43.75)	9 (47.37)
Data not Available	4 (20)	1 (5)

Assessment of circulating biomarkers in control and chest pain subjects

**Figure 1:**
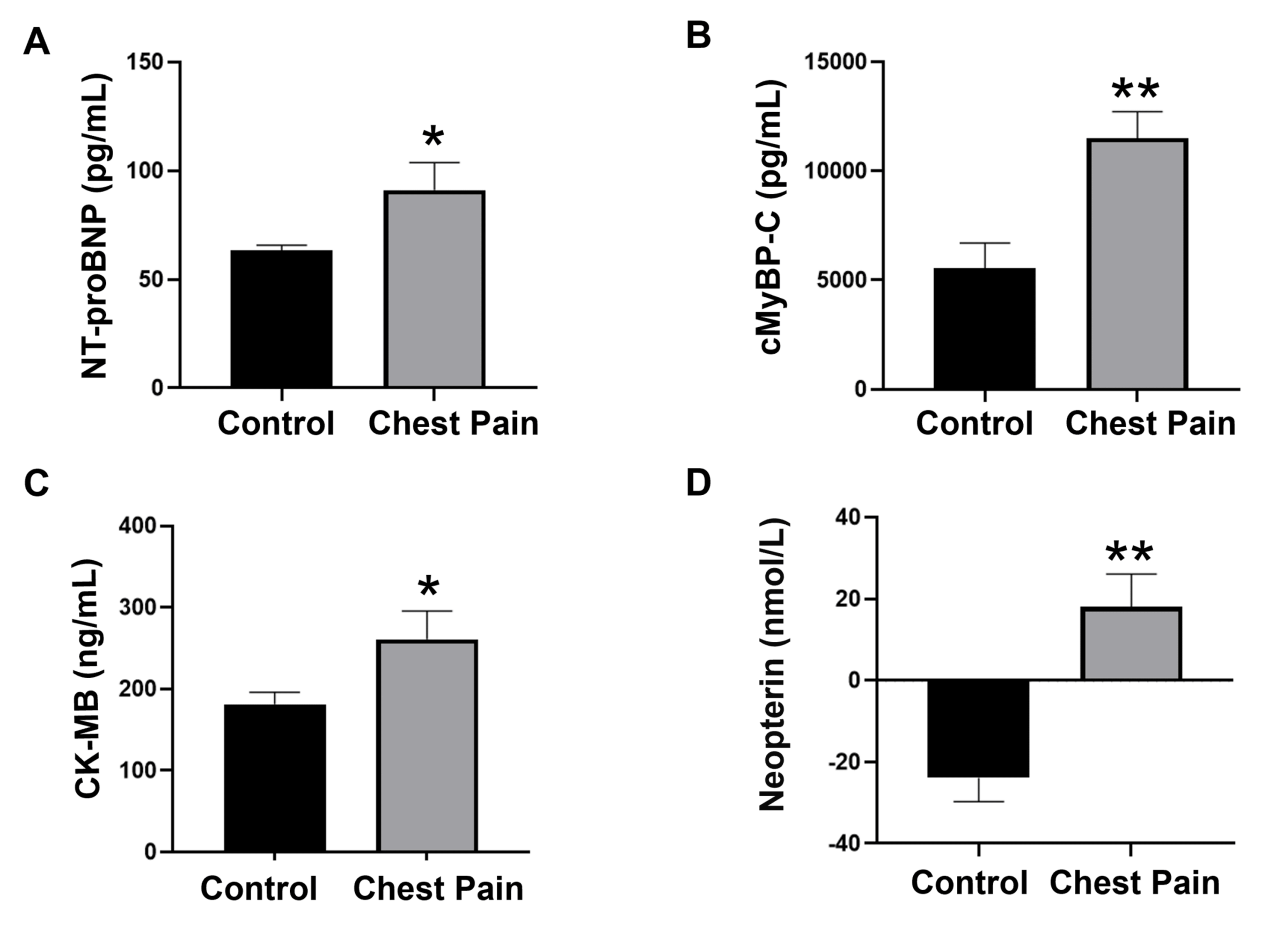
Quantitative analysis of plasma concentrations of (A) NT-proBNP, (B) cMyBP-C, (C) CK-MB and (D) Neopterin ELISA was performed to determine the concentration of key plasma biomarkers in the study population, in which a significant increase was observed in the chest pain cohort. Plasma concentrations of (A) NT-proBNP (n=20), (B) cMyBP-C (n=18-20), (C) CK-MB (n=13-15), and (D) Neopterin (17-19). Values represent mean ± SEM. *p<0.05, **p<0.01 vs. Control. NT-proBNP: N-terminal pro-B-type natriuretic peptide; cMyBP-C: cardiac isoform of myosin-binding protein C; CK-MB: creatine kinase-myocardial band; SEM: standard error of the mean

As blood-based biomarkers can be a useful tool to assess cardiac function, in the present study, we analyzed a panel of important biomarkers that are significantly related to cardiac disease pathology. The plasma level of NT-proBNP, the key biomarker of myocardial stress and left ventricular dysfunction, was significantly (p < 0.05) increased in the chest pain cohort compared to healthy subjects (Figure 1A). In addition, cMyBP-C, the marker of myocardial injury, showed a significant (p < 0.01) elevation in the plasma of the chest pain population as compared to the control cohort (Figure 1B). Similarly, compared to healthy subjects, the plasma levels of CK-MB, the marker of myocardial damage, showed a significant (p < 0.05) increase in the chest pain population (Figure 1C). One of the key markers of inflammatory progression in cardiovascular pathology, neopterin, also showed significantly (p < 0.01) higher levels in chest pain patients than in controls (Figure 1D). These results suggest the significant dysregulation of these important biomarkers in chest pain patients even in the absence of a definitive ACS diagnosis, supporting the additive value of biomarker profiling in chest pain evaluation.

Correlation analysis of plasma NT-proBNP with circulating levels of cMyBP-C, CK-MB, and neopterin in the study population

**Figure 2:**
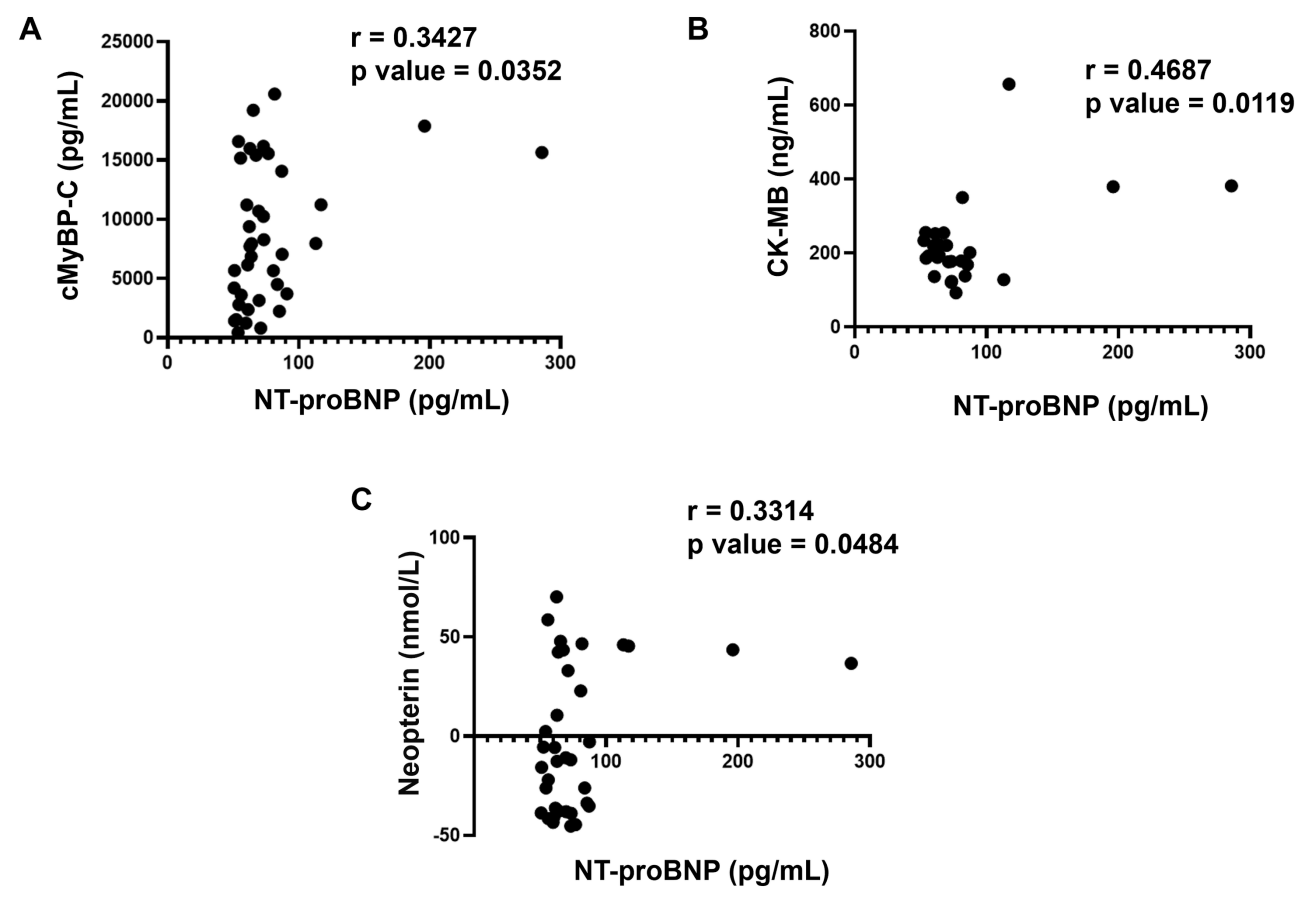
Correlation analysis of NT-proBNP with (A) cMyBP-C, (B) CK-MB and (C) neopterin Correlation was determined using Pearson’s r coefficient choosing two tailed P-value to demonstrate significance (alpha=0.05) and presented as scatter dot plot. Each plot independently shows corresponding correlation coefficient (r value) and significance (p value). NT-proBNP: N-terminal pro-B-type natriuretic peptide; cMyBP-C: cardiac isoform of myosin-binding protein C; CK-MB: creatine kinase-myocardial band

As NT-proBNP is an important and highly sensitive biomarker for the prognosis and early risk stratification of ACS, we evaluated the link between NT-proBNP and other circulating biomarkers analyzed in the present study. A statistical correlation analysis was performed, in which the extent of correlation was assessed in terms of Pearson’s r coefficient. Briefly, NT-proBNP showed a significant positive correlation with cMyBP-C, with r = 0.3427 (Figure 2A). Similarly, NT-proBNP and CK-MB were significantly correlated with r = 0.4687 (Figure 2B). Also, a significant positive correlation between NT-proBNP and neopterin was observed with r = 0.3314 (Figure 2C).

## Discussion

ACS is a major contributor to global morbidity, mortality, and healthcare expenditure, with chest pain being one of the most common reasons. Despite advances in diagnostic approaches, early and accurate identification of ACS by distinguishing cardiac from non-cardiac causes of chest pain remains challenging, especially during early presentation when clinical findings may be nonspecific. To reduce the future risk of irreversible myocardial damage, heart failure, and death, effective early risk stratification is essential, underscoring the prognostic importance of circulating biomarkers. In the present exploratory cross-sectional biomarker profiling study, we evaluated a panel of reliable circulating biomarkers that can be correlated with cardiac dysfunction in chest pain patients and emphasized their potential translational utility to enhance patient outcomes in a high-risk population.

Pro-B-type natriuretic peptide (proBNP) is secreted by myocardial cells in response to ventricular wall stress and further cleaved to form B-type natriuretic peptide (BNP) and its NT-proBNP [27,28]. They are closely linked to the regulation of cardiovascular homeostasis by regulating blood pressure, sodium balance, and extracellular fluid volume [29]. In addition, they modulate vascular smooth muscle tone, coronary blood flow, and proliferative responses during myocardial and vascular remodeling and regulate renin secretion, progesterone release, endothelin secretion, and vasopressin secretion [29-31]. It is well established that NT-proBNP is correlated with myocardial stress and left ventricular dysfunction in cardiac patients [32-37]. The structural and functional changes in the myocardium due to chronic pressure overload can be reflected in the increased NT-proBNP levels [38]. Studies have shown that neurohumoral activation, evidenced by the elevated levels of NT-proBNP in the circulation, is a powerful determinant of ACS for identifying early disease progression and future intensive care [27,36,39]. Of note, it has been used as a screening tool for cardiovascular complications in high-risk populations [40]. The observed elevation in the levels of NT-proBNP in the chest pain population of the present study is in line with these previous studies, linking adverse cardiac function and poorer clinical outcomes in these patients.

MyBP-C is an important sarcomeric protein that modulates cardiac function by regulating cardiac contractility, structural integrity, and relaxation [41]. It is a thick filament-associated protein that binds myosin, localized to the crossbridge-containing C zones in the myofibrils of all striated muscle [42]. Its cardiac isoform (cMyBP-C) consists of immunoglobulin modules and phosphorylation sites. The phosphorylation sites at the N terminus regulate cardiac contraction, and the C-terminal region binds to myosin rods for stabilizing the thick filament structure [43-45]. Evidence of cMyBP-C degradation under myocardial dysfunction, followed by its specific increase in the bloodstream, has been demonstrated in various studies [46-49]. The catalytic cleavage of cMyBP-C, followed by myocardial necrosis, releases its N-terminal fragment (C0-C1f) that further disrupts the interaction of native cMyBP-C with the thin filament for myosin-regulated contraction and leads to chronic cardiac dysfunction [50,51]. The high abundance and rapid release kinetics of cMyBP-C in response to myocardial injury offer potential diagnostic advantages in the early phases of ACS [21,52]. The chest pain cohort of our study also showed a significant increase in the level of cMyBP-C compared to healthy controls, emphasizing the possible cardiac damage in this population that warrants further investigation.

Creatine kinase (CK) is a critical enzyme involved in cellular energy metabolism of tissues with high energy demands, including skeletal muscle, cardiac muscle, and the brain [53,54]. CK-MB is the isoform that is primarily found in heart muscle cells, which in turn is released into circulation after myocardial injury and necrosis [55]. Various studies have shown the prognostic value of CK-MB in various cardiovascular pathologies, highlighting its importance in risk stratification [23,53,56-59]. In agreement with these studies, our chest pain population also showed increased CK-MB levels, suggesting possible myocardial damage and future adverse cardiac events that may offer additive prognostic value when interpreted along with other cardiac biomarkers using longitudinal outcomes.

Neopterin, a by-product of the guanosine triphosphate-biopterin pathway, is synthesized by macrophages and released into circulation in response to inflammation and immune system activation [60]. It is produced in macrophages after the induction of guanosine triphosphate (GTP) cyclohydrolase I by Interferon-gamma (INF-γ) and thus any condition that leads to the activation of INF-γ can induce production of neopterin [60,61]. Studies have shown that increased neopterin levels can be correlated with various adverse cardiac events [22,62-64]. The chronic inflammatory process and macrophage activation can lead to acute clinical events in ACS by the induction of plaque rupture, which in turn triggers the neopterin release into the circulation [63]. As inflammation and immune activation are the early events in cardiac dysfunction, elevated levels of neopterin have been used as a promising circulating biomarker for risk stratification in cardiac diseases [63-68]. The activation of inflammatory signaling and possible cardiac dysfunction in our chest pain population was evidenced by the increased levels of neopterin in this population as compared to healthy subjects. These elevated levels of neopterin may reflect the heightened immune activation in patients at increased risk of cardiovascular dysfunction and suggest the role of inflammatory biomarkers in the risk stratification, along with markers of myocardial injury and dysfunction.

Systematic statistical correlation analysis of multiple biomarkers integrates various pathophysiological pathways, reflecting disease heterogeneity and improved diagnostic potential [69]. Early identification of cardiac dysfunction in ACS remains a major clinical challenge because of the heterogeneous pathologies in ACS progression. Hence, to evaluate the interdependence and combined diagnostic potential, we have performed the correlation analysis of NT-proBNP with other biomarkers tested. We observed a positive correlation of NT-proBNP with other cardiac biomarkers such as MyBP-C and CK-MB, suggesting a significant association between structural myocardial stress, damage, and necrosis. A positive correlation between NT-proBNP and neopterin underscores the involvement of immune and inflammatory signaling pathways in myocardial stress that leads to ACS progression. Thus, the correlation demonstrated between these biomarkers in our study population provides a multidimensional view of cardiac dysfunction characterized by myocardial wall stress, ventricular dysfunction, myocardial injury, immune activation, and myocardial necrosis that may lead to ACS. The utilization of the multimarker superiority of the proposed biomarkers should be verified using detailed statistical analysis, such as receiver operating characteristic (ROC) analysis, area under the curve (AUC) calculation, Net reclassification improvement (NRI), and Integrated discrimination improvement (IDI).

Clinical significance and limitations of the study

**Figure 3:**
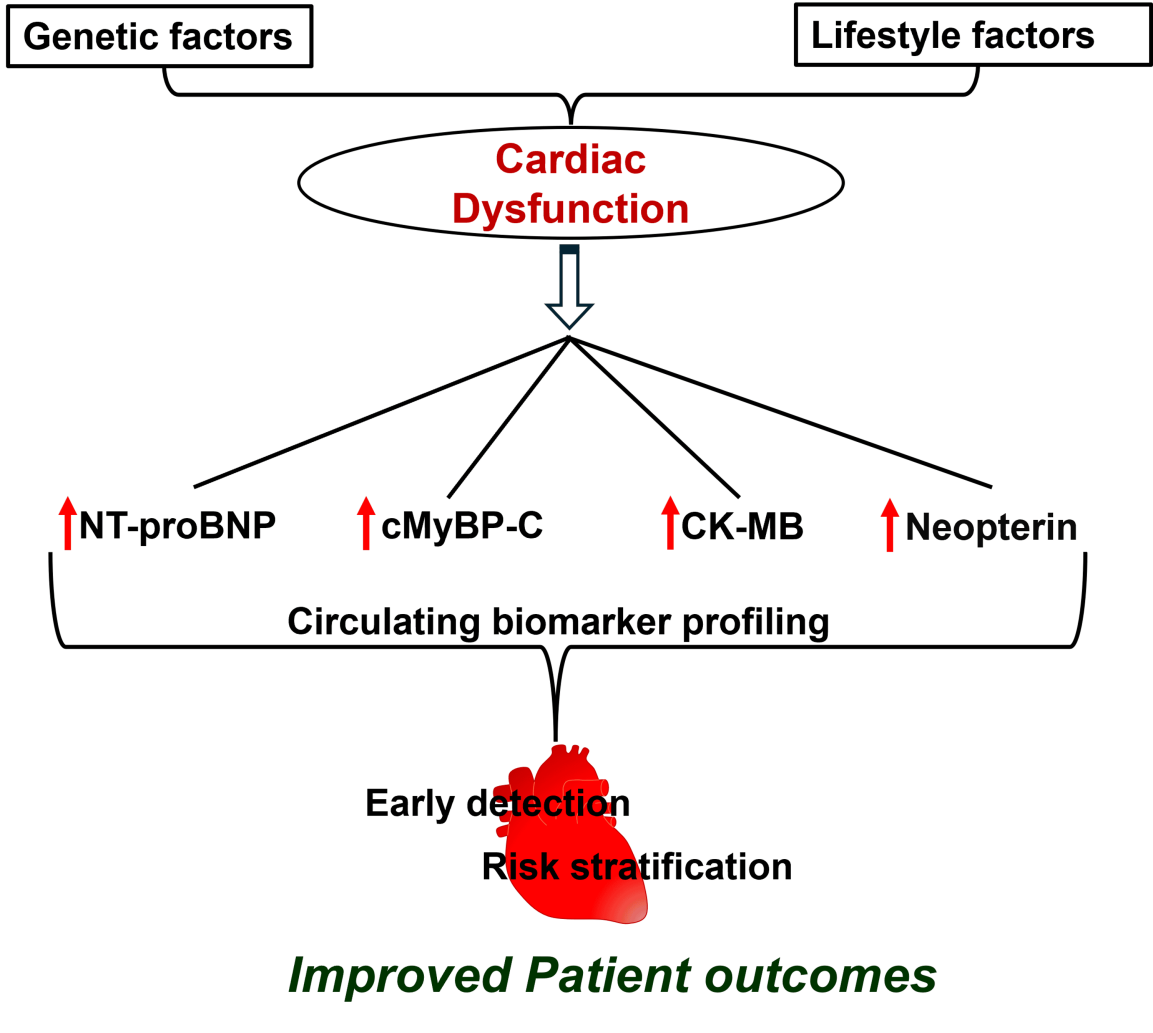
Schematic representation showing the importance of panel of biomarkers profiling for the risk stratification of cardiac causes for chest pain The interplay between genetic predisposition and lifestyle factors that contribute to cardiac dysfunction results in the dysregulation of key biomarkers, which can be detected in plasma using an integrated biomarker approach. Thus, the study highlights the potential utility of selected biomarkers as a lead for future studies to explore early detection, risk stratification, and improved patient outcomes in chest pain patients. NT-proBNP: N-terminal pro-B-type natriuretic peptide; cMyBP-C: cardiac isoform of myosin-binding protein C Image Credit: Authors

The findings of the present study have clinical implications that suggest a panel of biomarkers to provide better assessment of cardiac dysfunction in chest pain patients than individual markers alone, as summarized in Figure 3. The study suggests the importance of future follow-up of these patients, and in the event of any abnormal findings, detailed cardiac clinical characterization can be performed to prevent irreversible cardiac damage. The study underscores the potential clinical utility of the studied biomarker panel in cardiovascular risk prediction in the future after detailed longitudinal studies, and the incorporation of these into clinical practice may help to optimize decision-making and therapeutic management in ACS. Early screening and timely diagnosis are essential to manage the progression of irreversible cardiac damage and chronic disease progression, especially in the high-risk rural population of West Virginia, where access to advanced healthcare facilities is limited.

However, the study has some limitations that warrant consideration. The relatively small sample size, consisting of a homogenous population of the study, limits the generalizability of the findings to broader populations. Detailed biomarker correlation modeling using a large population size and consideration of other relevant confounding variables is crucial for the translational applicability in clinical settings. Additionally, the present cross-sectional design limits evaluation of longitudinal outcomes such as subsequent cardiovascular events or mortality. Detailed statistical analysis and systematic risk prediction modeling are warranted to establish the prognostic utility of the studied panel in ACS risk stratification.

## Conclusions

The present pilot study demonstrates a panel of circulating biomarkers consisting of NT-proBNP, MyBP-C, CK-MB, and neopterin, which are shown to have a significant increase in non-ACS patients with chest pain compared to healthy subjects. The correlation analysis explored the interrelationship between these markers, which gives additive information about the diverse pathophysiological changes in cardiac dysfunction that may progress to ACS. Hence, the findings of the present study support the future translational potential of a multi-biomarker approach for early detection, risk stratification, and therapeutic interventions of the cardiac causes of ACS detection, with the eventual goal of improving patient outcomes and reducing the healthcare burden associated with ACS in the rural population of West Virginia. Future research involving larger, heterogeneous cohorts with longitudinal follow-up is warranted to validate the prognostic utility of the biomarker panel in clinical settings for the early ACS detection and risk stratification.
